# Promoting simulation-based training in radiology: a homemade phantom for the practice of ultrasound-guided procedures

**DOI:** 10.1259/bjr.20220354

**Published:** 2022-08-03

**Authors:** Elisabetta Giannotti, Ketan Jethwa, Samantha Closs, Rachel Sun, Hamnah Bhatti, Jonathan James, Christopher Clarke

**Affiliations:** Nottingham Breast Institute, Nottingham University Hospitals NHS Trust, Nottingham City Hospital, Hucknall Road, Nottingham, UK; University Hospitals of Derby and Burton NHS Foundation Trust, Royal Derby Hospital, Uttoxeter New Road, Derby, UK; Nottingham Breast Institute, Nottingham University Hospitals NHS Trust, Nottingham City Hospital, Hucknall Road, Nottingham, UK; Nottingham Breast Institute, Nottingham University Hospitals NHS Trust, Nottingham City Hospital, Hucknall Road, Nottingham, UK; Department of Clinical Radiology, Nottingham University Hospitals NHS Trust, Queens Medical Centre, Derby Road, Nottingham, UK; Nottingham Breast Institute, Nottingham University Hospitals NHS Trust, Nottingham City Hospital, Hucknall Road, Nottingham, UK; Department of Clinical Radiology, Nottingham University Hospitals NHS Trust, Queens Medical Centre, Derby Road, Nottingham, UK

## Abstract

**Objective:**

Ultrasound-guided intervention is an essential skill for many radiologists and critical for accurate diagnosis and treatment in many radiology subspecialties. Simulation using phantoms have demonstrated statistically significant benefits for trainees within the literature. We propose a novel phantom model which the authors feel is ideal for training clinical radiology trainees in the performance of ultrasound-guided procedures.

**Methods:**

The recipe to prepare a homemade phantom is described. Results of a local survey from trainees preparing and using the phantom are also presented.

**Results:**

This realistic training simulation model can be adapted to suit a variety of biopsy devices and procedures including soft tissue biopsy and cyst aspiration. The phantom mimics the sonographic appearances of soft tissue and biopsy targets can be concealed within. The phantom was easily prepared by 22 trainees (Likert score 4.5) and it functioned well (Likert score of 4.7).

**Conclusion:**

In summary, our phantom model is ideal for training clinical radiology trainees in the performance of ultrasound-guided core biopsy. The availability and low cost of the model, combined with the ease of preparation and reproducibility, make this an efficient and effective addition to the training process.

**Advances in knowledge:**

A low cost easily handmade phantom recipe is described that could be easily implemented in training schemes.

The proposed phantom mimics soft tissue with targets concealed within it and allows for repeat interventions.

## Background

Ultrasound is utilised by radiologists for both diagnostic and interventional procedures, and ultrasound guidance has become the standard of care for many common interventional procedures, such as core needle biopsy, fluid aspiration, abscess drainage and vascular access, providing real-time visualisation of the needle during the procedure. This technique requires practice for trainees to develop hand–eye coordination and psychomotor abilities to ensure a safe approach and visualisation of the needle. In many centres, trainees learn ultrasound-guided procedures by performing these on patients under direct supervision.

In recent years, simulation has played a greater role in training and when integrated into a curriculum, simulation-based training can supplement and enhance the traditional apprenticeship model of teaching by reducing training variability and offering a more standardised educational approach.^1^ An important benefit is improving patient safety by allowing trainees to practise without harming patients.^2^

This has prompted the development of phantoms for use in radiology training. Using a phantom allows trainees to gain familiarity with targeted ultrasound-guided procedures in a safe environment,^3^ and has been shown to improve technical procedural skills,^1,2,4,5^ reduce anxiety,^1^ improve confidence reduce the risk of potential complications^6^ and improve the proficiency of novices undertaking ultrasound-guided procedures.^2,7–15^

Unfortunately commercially available ultrasound phantoms are limited by their high cost and may degrade with repeated use. Alternatives such as raw meat work well as phantoms, but contain high levels of bacteria that can represent a health risk when used in clinical environment with ultrasound machines also used for patient care. We have developed an ultrasound phantom which closely mimics the sonographic appearances of soft tissue and is made from hygienic and inexpensive materials.

## Methods and materials

The phantom is gelatine-based with a corn flour additive and constructed using widely materials found in a typical supermarket at a cost per phantom of less that £10. The targets for intervention are either solid or cystic and distributed throughout the gelatine body. There are simulated cysts made from a small balloon, or the finger of a disposable glove filled with water. Solid targets for core biopsy are created using olives stuffed with raisins. Stuffing the olives with different numbers of raisins produces creates different weights ensuring that targets will sit at different depths within the phantom when the gelatine sets. Each trainee makes their own phantom before attending their interventional procedures training session.

## Constructing the phantom

Equipment required: bowl, tablespoon, saucepan, plastic food container (preferably rectangular), cling film/plastic wrap, tray.

### Ingredients

- 1000 ml hot water from the tap
- 160 g corn flour
- 5 sachets of powdered gelatine (or vegan alternative)
- food colouring (optional)
- 300 g of tofu in 1–2 cm thickness slices
- 5–10 pitted olives (targets)
- raisins
- cyst phantom (small water balloon or a finger from a disposable glove filled with water).

Preparation time: 20 min

Setting time: 12 h

### Instructions are as follows

1. Mix corn flour and hot water in a saucepan until dissolved and contents are mixed well. Using a whisk may help. Adding food colouring is optional but helps in hiding the targets inside the phantom.
2. Gently heat the mixture of corn flour and water whilst stirring continuously and add the gelatine (or alternative). Stir until the liquid thickens to the consistency of “pannacotta”.
3. Remove from the heat.
4. Prepare the targets.
  1. Olives stuffed with 1–3 raisins
  2. Small balloon or disposable glove finger filled with water.
  3. Further targets options include dried apricots or prunes and peppercorns that can mimic calcifications.
5. Line the plastic container with cling film and pour a small amount of the gelatine mixture into the bottom. Place tofu (of 1 cm depth) over the bottom covering the whole area, preferably without a gap between the pieces, as the tofu layer is intended to mimic skin.
6. Pour over the remaining gelatine and corn flour mixture. Carefully place the targets within the hot liquid utilising a spoon (the viscosity of the liquid will prevent the olives from being displaced).
7. Allow to cool at room temperature (approximately 30 min) and then set in the refrigerator for a minimum of 12 h. Keep refrigerated until use.
8. The phantom should fall easily from the container when inverted. It should be placed on a tray to prevent accidental damage to the underlying surface (typically the patient couch) during biopsy practice sessions. Remove the cling-film and the phantom is ready for use.

Disposal: the phantom can be disposed with food waste.

## User survey and feedback

We have been using this phantom in our department locally for the last 5 years for every radiology trainee undertaking core breast training prior to starting biopsy work on patients and the recipe has been developed over time. Feedback regarding the effectiveness of the phantom in boosting trainee confidence and biopsy skills has anecdotally been positive; however, we did not have formal data on this. therefore questions assessing the ease of phantom preparation and its effectiveness as a training tool were included as part of an overall course feedback questionnaire for a larger interventional radiology skills course for second year radiology trainees utilising the phantoms for biopsy training. Responses were sort and scored using a 5-point Likert scale: (1) Strongly disagree; (2) Disagree; (3) Neither agree nor disagree; (4) Agree; (5) Strongly agree.

## Results

### Phantom characteristics

**Figure 1.**
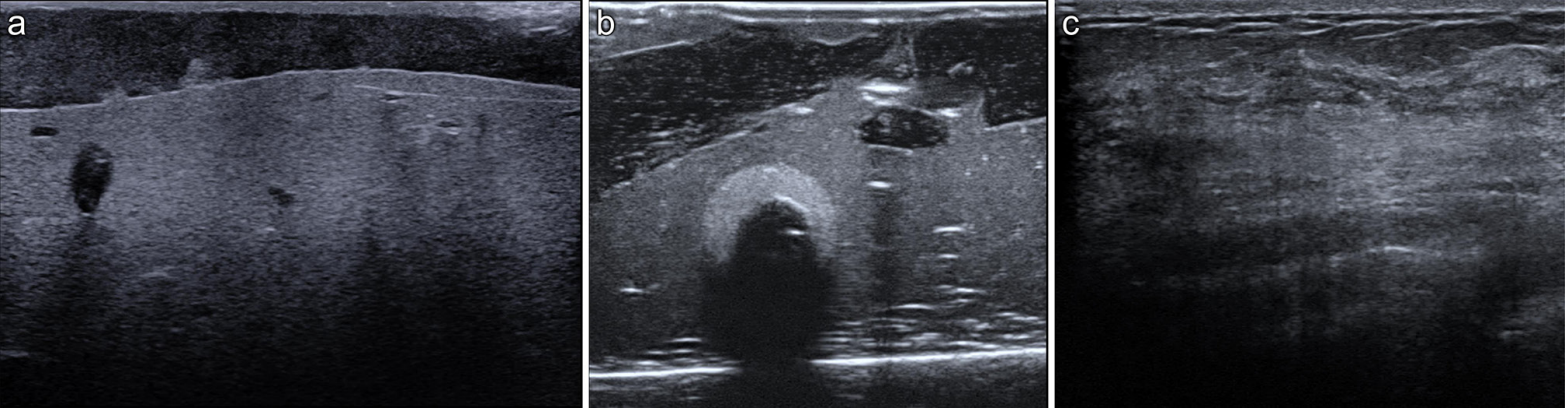
Comparison of ultrasound echotexture of the phantom (1a) and phantom with olive (1b) with human breast tissue (1c).

**Figure 2.**
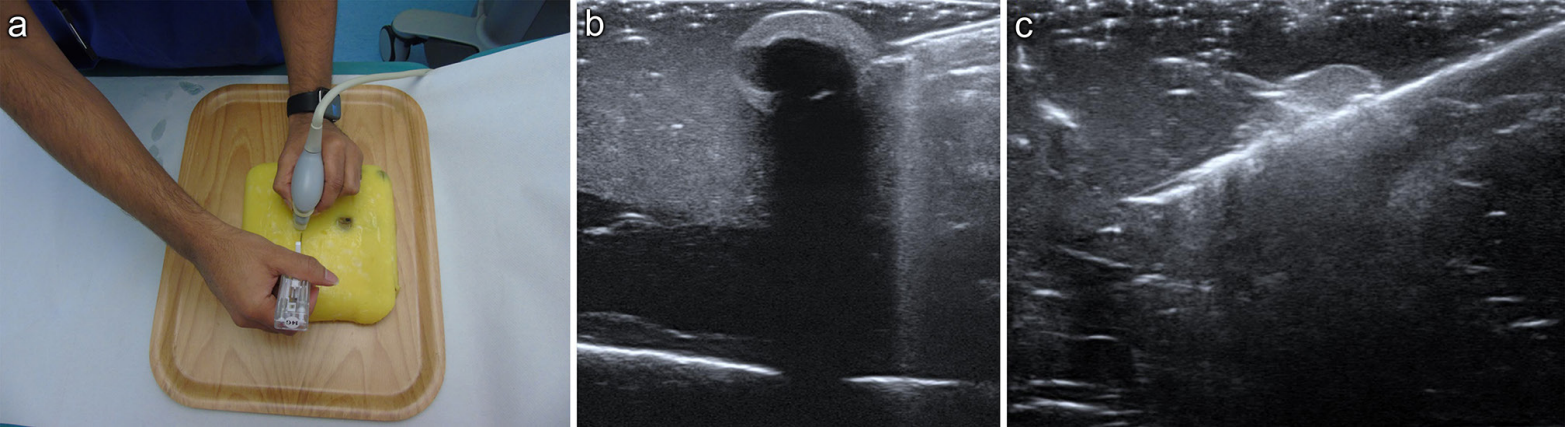
Using this phantom enables training in all practical aspects of performing ultrasound-guided biopsy including needle-probe position (2a), visualising the target (2b) and visualising the needle passing through the target during a biopsy (2c).

**Figure 3.**
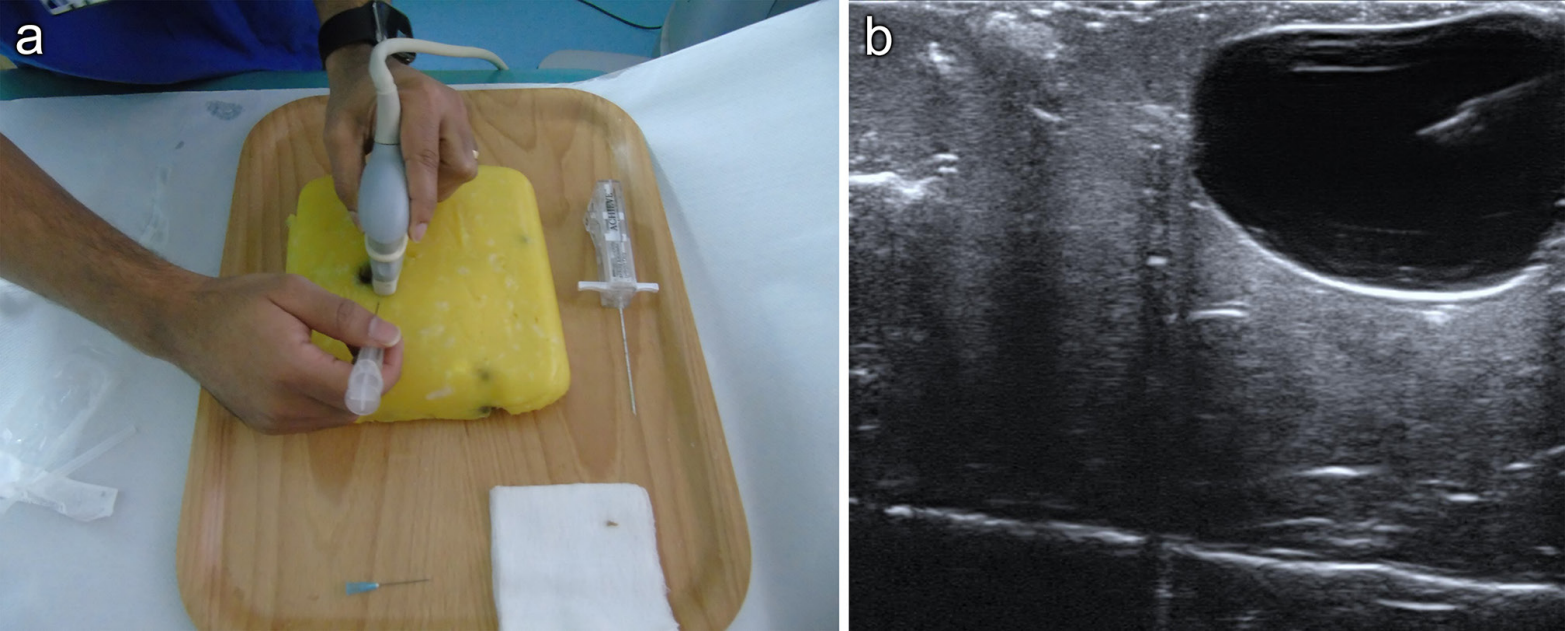
Using this phantom to undertake ultrasound-guided cyst aspiration (3a). The needle is clearly visualised within the cyst and fluid can be aspirated under ultrasound guidance (3b).

The ultrasound appearances of the phantom (Figure 1a and b) are very similar to human breast tissue (Figure 1c). The size and shape of the phantom can be easily varied by choosing containers of appropriate size and shape. The introduction of tofu on top of the phantom mimics the skin and makes the phantom more resistant to fracturing during the practice, particularly if pressure is applied. The phantom can be used repeatedly, provides tactile feedback with a similar consistency to human tissue and will hold a needle in place and not generate an obvious needle track. Using this phantom enables practical training in performing ultrasound-guided biopsy (Figure 2) and cyst aspiration (Figure 3). The targets can be visualised and then localised with a needle under ultrasound guidance. When undertaking core biopsy practice, direct visual inspection that the biopsy sample contains both olive and raisin confirms accurate targeting by confirming that the centre, as opposed to just the periphery of target has been sampled. Inserting 3–5 ml of ultrasound gel into the phantom can also mimic an abscess drainage.^16^

The phantom will eventually fragment, but this is typically after around 30 passes of the biopsy needle. This is sufficient to allow for training 1–2 people, and multiple phantoms can be prepared if required.

### Experience from our centre

22 trainees who had created the phantom completed the survey of user experiences. In response to the question “I found it easy to follow the instructions to create the ultrasound phantom”, a Likert score of 4.5 was achieved (4 – Agree and 5 – Strongly Agree with the statement). The trainees were easily able to follow the instruction to create their own phantom (Likert score 4.5). In the response to the question “the ultrasound phantom functioned well”, a Likert score of 4.7 was achieved (Table 1).

**Table 1.** Results from a simple questionnaire answered by 22 second year radiology trainees as part of a local interventional radiology skills course

QUESTION	Average score on Likert scale 1 (strongly disagree) to 5 (strongly agree)	Number of trainee responses
“I found it easy to follow the instructions to create the ultrasound phantom biopsy model”	4.5	22
“The ultrasound phantom functioned well to demonstrated interventional techniques”	4.7	22

## Discussion

Trainees found our gelatine and corn flour-based phantom easy to construct and use in simulation-based training. Characteristics that define a good phantom include similar echogenicity to human tissue, readily available components, and low cost.^15^ Our phantom is based on gelatine and so low cost at less than £10 per phantom. Others have also described the production and use of homemade gelatine phantoms.^8,17–22^

Phantoms can be categorised into four main groups: water-based, commercial, meat and gelatine-based. Water phantoms do not mimic biological tissue making it less than ideal for beginners practicing needle placement as they do not provide tactile feedback and water cannot hold a needle in place.^15^ Commercial phantoms are typically produced from hydrogel polymer or elastomer rubber.^23–25^ Most importantly, they are expensive (approximatively $400 USD^16^ and so are unaffordable for many training centres and trainees. They may also degrade after repeated usage. They tend to have an overly firm texture, and so may not provide realistic tactile feedback to the user. The ultrasound appearances also do not closely match human tissue as they tend to exhibit uniform echogenicity and so could lead to false confidence in performance as a needle is more easily identifiable. In addition, it is difficult to incorporate a target within the structure, such as a fluid collection.^15^ Meat phantoms, *e.g.* turkey or chicken breast, are relatively cheap, can give tactile feedback and have an echogenicity which mimics human tissue but have the disadvantage of potential bacterial contamination.

Variations of gelatine phantoms have been described in the literature to improve performance. The addition of corn flour results in ultrasound echogenicity similar to soft tissue^26^ and so this is the approach we adopted. Gelatine-only phantoms are more sonolucent and so the needle may appear more echogenic and so this may make biopsies artificially easier to perform due to improved needle visualisation.^19^ The addition of corn flour provides a truer reflection of the in-vivo situation. Corn flour also renders the phantom opaque hiding the biopsy needle target from external view. The addition of tofu on the surface of our phantom has not been described previously but has the advantage of mimicking skin and prolongs the life of the phantom during multiple biopsy attempts. Alternatives to the corn flour additive have been tried. Psyllium husk has also been found to increase opacity and background echogenicity and produce sufficient tactile feedback for practising needle handling.^18^ However, Psyllium husk is not stocked in most supermarkets. Similarly, Mung bean starch has been advocated as an additive to gelatine, but again is not readily available.^6^

There are alternatives to the use of food grade gelatine. Gelatine-agar,^27^ paraffin-gel wax,^28^ PVC^28–31^ and silicone rubber-based^32^ phantom have all been reported as a base material for ultrasound phantoms with superior acoustic and mechanical properties.^22^ However these phantoms require a more complex fabrication process mixing multiple materials, long preparation and setting times and high temperature heating (typically 180–200°C). The cost of materials is also increased at around $60 USD per phantom.^22^

In conclusion, our gelatine phantom is low cost, hygienic and uses readily available ingredients. The addition of corn flour makes the phantom echogenicity very similar to human soft tissue. It is easy to produce and receives excellent trainee feedback and so provides an efficient and effective phantom for simulation training of ultrasound-guided procedures.
